# Advances in photothermal-driven tumor immunomodulation and synergistic therapy

**DOI:** 10.3389/fimmu.2026.1952895

**Published:** 2026-10-23

**Authors:** Xiuyun Wei, Changjiang Mo, Jiacheng Chen, Deli Li, Qinting Wu, Shenxia Xie, Xiaomei Yang

**Affiliations:** 1 School of Basic Medical Science, Guangxi Medical University, Nanning, Guangxi, China; 2 Second Clinical Medical School, Guangxi Medical University, Nanning, Guangxi, China; 3 Guangxi Key Laboratory of Nanobody Research; Guangxi Nanobody Engineering Research Center, Guangxi Medical University, Nanning, Guangxi, China; 4 School of Pharmacy, Guangxi Medical University, Nanning, Guangxi, China

**Keywords:** combination therapy, immunotherapy, photothermal materials, photothermal therapy, tumor

## Abstract

Photothermal therapy (PTT) is a local treatment that uses photothermal conversion agents (PTAs) to absorb light and produce heat in tumor tissue. The direct result is tumor ablation. At the same time, heat-damaged tumor cells may undergo immunogenic cell death (ICD). This process is often accompanied by the release of damage-associated molecular patterns (DAMPs), followed by dendritic cell activation and antigen presentation. Because of this immune effect, PTT has been explored as a partner for immunotherapy. It may be useful in tumors where antigen presentation is weak or where the immune microenvironment is highly suppressive. However, local heating alone usually cannot produce a long-lasting systemic immune response. Its effect on distant metastasis is also limited. For this reason, PTT is now often combined with immune checkpoint blockade, adoptive cell therapy, cancer vaccines, and other immune-based strategies. This review discusses the link between PTT-induced tumor damage and antitumor immune activation. It classifies and compares the structural optimization ideas, optical properties, biological safety, and application limitations of organic and inorganic photothermal materials. It summarizes the six mainstream combination schemes of photothermal therapy with immune checkpoint inhibitors, adoptive cell therapy, tumor vaccines, cytokine therapy, oncolytic virus therapy, and bispecific antibody therapy. It analyzes the synergistic anti-tumor mechanisms and the latest preclinical research progress of various combination strategies. Finally, it sorts out the core bottlenecks that restrict the clinical transformation of photothermal immunotherapy and proposes corresponding optimization development prospects.

## Graphical Abstract

**Figure:** This review summarizes recent advances in the integration of PTT with various immunotherapeutic approaches, including immune checkpoint inhibitors, adoptive cell therapy, cancer vaccines, cytokine therapy, oncolytic virus therapy, and bispecific antibody therapy (**Figure 1**).

## 1 Introduction

Cancer is still a major health problem worldwide. Prevention and early diagnosis have improved, but new cancer cases continue to rise in many regions (1). Surgery, radiotherapy, and chemotherapy are still widely used in clinical practice. Their effects, however, are different among cancer types and stages. Surgery works well for many localized tumors, but it is much less useful after the cancer has spread. Damage to nearby normal tissues can also occur during surgery (2). Radiotherapy has a similar problem. For some tumors, radiotherapy provides good local control, although nearby normal tissues can also be exposed during treatment. The response is also poor in some types of cancer (3). Chemotherapy is different because the drugs circulate throughout the body and can therefore act on tumors at more than one site. This is useful when the disease has spread, but toxicity remains a problem. Another difficulty is that cancer cells may gradually become resistant to the drugs being used (4). As a result, treatment of metastatic and recurrent cancer remains difficult, and alternative therapeutic approaches are still being investigated.

**Figure 1:**
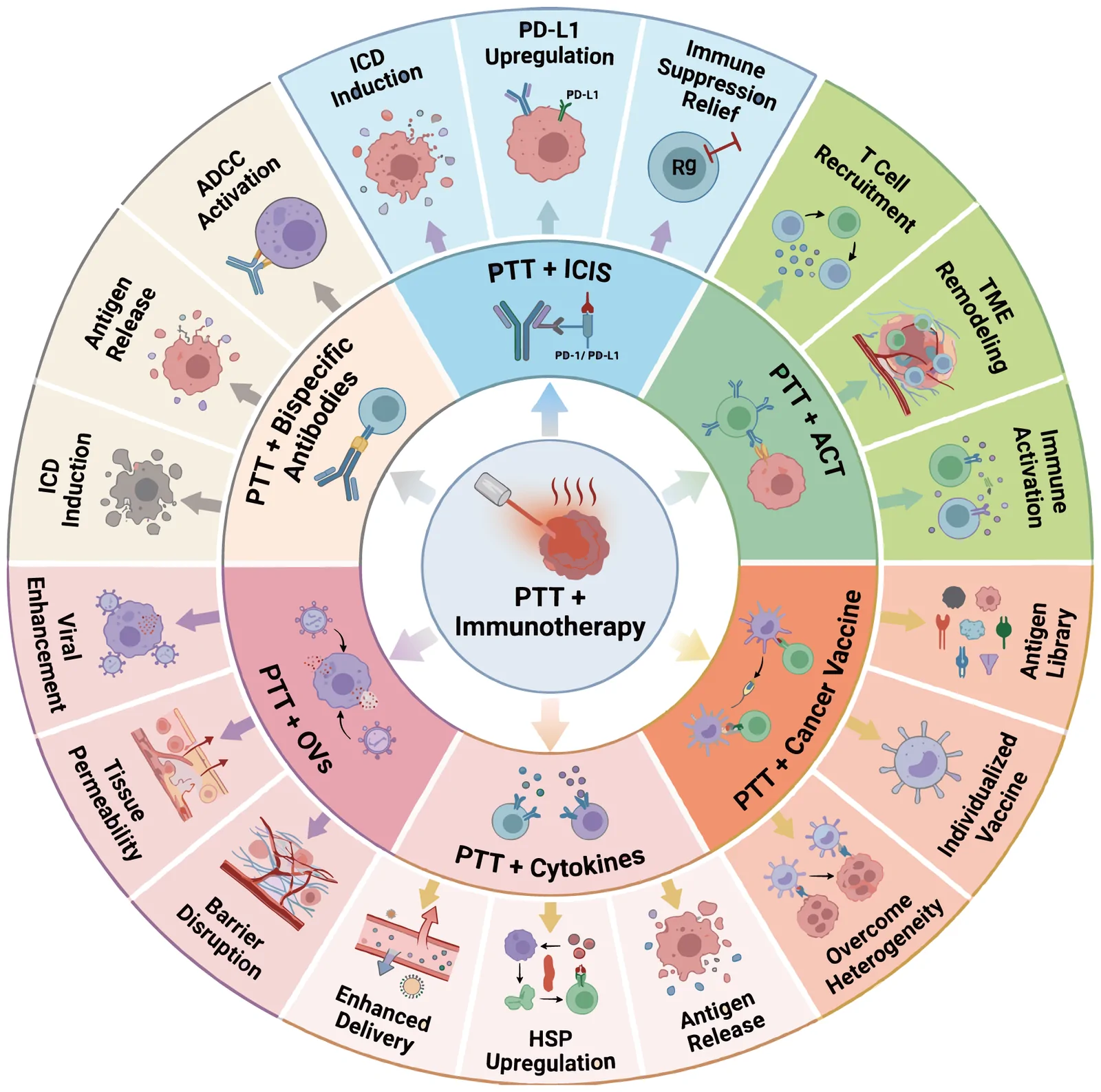
Schematic illustration of the synergistic mechanisms underlying photothermal therapy combined with various immunotherapeutic strategies for tumor treatment.

Photothermal therapy (PTT) is a minimally invasive approach that uses light-responsive agents to generate heat within tumor tissue. After absorbing light, photothermal agents (PTAs) convert the energy into heat, leading to local temperature elevation and, when sufficient, tumor cell apoptosis or necrosis. One reason PTT has received increasing attention is that its effect may not be limited to direct tumor killing. Thermal injury can also promote the release of tumor-associated antigens and damage-related signals, which may contribute to subsequent immune activation. This has provided a rationale for combining PTT with immunotherapy. Because heating can be confined to the irradiated area, PTT may also reduce systemic exposure compared with conventional chemotherapy (5).

The performance of PTT, however, depends on several practical factors. Light does not penetrate deeply into tissue, which limits its use for tumors located far from the body surface. Treatment can also be weakened when too little PTA reaches the tumor. In addition, heat shock proteins may be induced after thermal stress and help tumor cells tolerate subsequent heating (6). Temperature control is therefore important, since insufficient heating may fail to kill tumor cells, whereas excessive heating may injure surrounding tissues. Current studies have tried to address these issues by improving NIR absorption, increasing photothermal conversion efficiency, enhancing tumor accumulation of PTAs, and adjusting irradiation conditions. Combination with immunotherapy has become one of the main directions being explored, particularly because PTT may provide both local tumor destruction and immune-related effects.

Immunotherapy works by activating or reshaping the host immune system, including immune checkpoint inhibitors, adoptive cell therapy, cancer vaccines, and related approaches. In solid tumors, the effect of treatment is often limited by the tumor microenvironment (TME). Such as, poor immune cell infiltration, physical barriers, and low tumor immunogenicity reduce treatment efficacy (7). In hepatocellular carcinoma (HCC), for example, dysfunctional tumor-infiltrating T cells and checkpoint-mediated immune tolerance, including the PD-1/PD-L1 pathway, can limit antitumor immunity. This limitation gives a clear reason to combine immunotherapy with other treatments (8). In HCC, the combination of locoregional therapy and immunotherapy has already become an active clinical direction. Recent expert consensus also supports the use of immune checkpoint inhibitor-based combinations, including strategies that pair locoregional treatment with immunotherapy (9).

These findings provide a basis for exploring PTT together with immunotherapy. The synergy between PTT and immunotherapy is mainly related to three processes, as shown in **Figure 2**. First, PTT induces immunogenic cell death (ICD), and leads to the release of tumor-associated antigens and damage-associated molecular patterns (DAMPs). Then, these signals support dendritic cell maturation and T cell activation. Second, PTT may reshape the TME and improve immune cell infiltration and immune responsiveness. Finally, photothermal stimulation may increase the expression of immune checkpoint molecules, which could make tumors more sensitive to immune checkpoint inhibitors (10–12).

**Figure 2:**
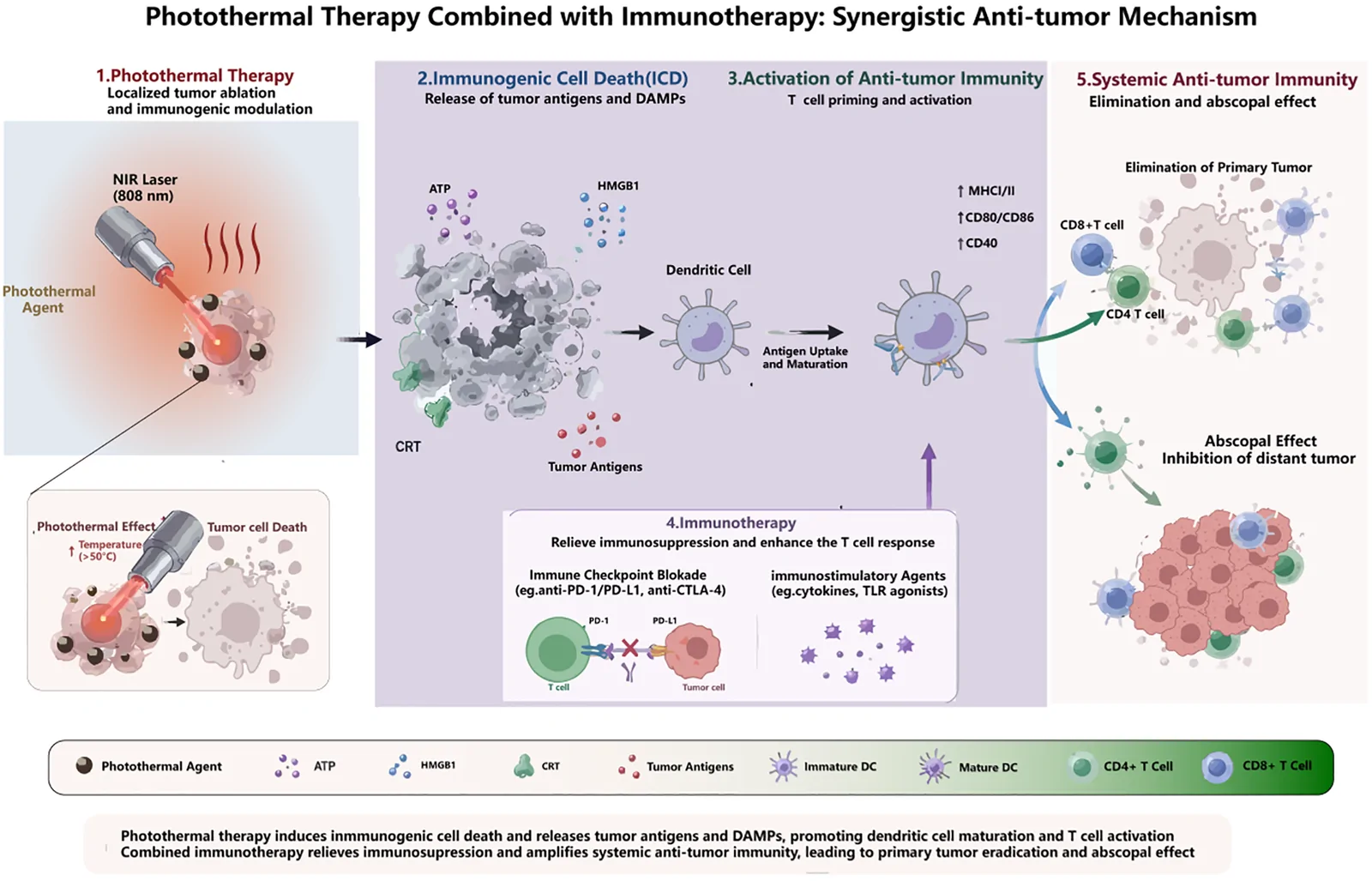
Schematic illustration of the synergistic antitumor mechanism of combined photothermal therapy and immunotherapy.

This review summarizes the fundamental principles of PTT, recent advances in the design and optimization of photothermal materials, current strategies for combining PTT with immunotherapy and the major challenges and future perspectives in this rapidly evolving field.

## 2 Photothermal therapy: mechanisms and photothermal agents

### 2.1 Mechanism of PTT

PTT works by heating the tumor after light exposure. This requires photothermal agents (PTAs), which absorb near-infrared light and convert it into heat. Melanin and hemoglobin can provide this effect naturally, while other PTAs need to be introduced into the tumor. The temperature reached during treatment varies with several experimental conditions, including the amount of PTA and the irradiation setting. Mild heating is usually associated with hyperthermia, whereas stronger heating can directly damage tumor cells. Temperatures above about 42 °C are often sufficient to produce substantial thermal injury (13). Damage to normal tissue depends largely on how well the heating is confined to the tumor region (14).

Cell death after PTT does not follow a single pathway. Stronger heating can disrupt the plasma membrane quickly and push cells toward necrotic death. Once membrane integrity is lost, intracellular molecules spill into the surrounding tissue, including DAMPs that can contribute to local inflammation and alter the tumor microenvironment. Apoptosis is more commonly associated with milder thermal stress. Early in this process, the plasma membrane remains intact, while phosphatidylserine appears on the cell surface and marks the dying cell for phagocytic clearance. If these cells are removed efficiently, little intracellular material is released. When clearance is delayed, membrane integrity may eventually be lost, leading to secondary necrosis and the release of DAMPs and other intracellular components (15).

To reduce unwanted inflammation and favor apoptosis over necrosis, researchers have adjusted the main PTT conditions, including irradiation power, exposure time, and the dose and type of PTAs. These factors affect both the safety and the antitumor effect of PTT. Wavelength is another important issue. Longer wavelengths usually penetrate tissue better (16). For this reason, near-infrared (NIR) light, especially 700-1400 nm, is widely used in PTT because it reaches deeper tissue and causes less photodamage than visible or ultraviolet light (17). The NIR region is often divided into NIR-I (700-950 nm) and NIR-II (1000-1700 nm) windows (18). NIR-I irradiation, particularly around 808 nm, is still the most commonly used light source in PTT studies. Most PTAs discussed in this review are based on NIR-I irradiation. By contrast, NIR-II irradiation, often represented by 1064 nm, has received growing attention because of its deeper penetration and lower light scattering. This makes it a promising option for deep-seated tumors. Therefore, although the following discussion primarily focuses on NIR-I-responsive PTAs in accordance with the predominant literature covered in this review, the distinct advantages and emerging applications of NIR-II-responsive PTAs should also be recognized (19). The selection and engineering of PTAs are also critical determinants of therapeutic performance. Ideal PTAs should possess high photothermal conversion efficiency, strong NIR absorption, favorable biocompatibility, low toxicity, and appropriate physicochemical properties, including particle size and stability (20). Accordingly, precise optimization of key treatment parameters, such as laser power density and irradiation duration, is essential for maximizing therapeutic outcomes (21). Furthermore, a deeper understanding of the molecular mechanisms underlying PTT-induced cell death may facilitate the development of strategies that reduce secondary inflammatory damage and further enhance treatment efficacy (22). These physicochemical criteria have guided the rational design of diverse PTAs (23), which can be broadly classified into organic and inorganic systems, as discussed in Section 2.2.

### 2.2 Photothermal agent

PTAs are the key functional components of PTT. When exposed to external light irradiation, particularly NIR light, PTAs convert absorbed photon energy into heat through non-radiative energy dissipation, thereby increasing the temperature of the target tissue (24). An ideal PTA should exhibit strong NIR absorption, high photothermal conversion efficiency, effective tumor-targeting capability, low toxicity, and excellent biocompatibility (25). In recent years, extensive efforts have been devoted to the development of novel PTAs, particularly nanoscale materials, which can be generally divided into two categories: organic PTAs and inorganic PTAs (see **Table 1**).

**Table 1:** Summary table of photothermal conversion agents in photothermal therapy.

PTA type	Category and name	Composition	Size (nm)	Irradiation condition	Injection dose	Achieved temperature (°C)	Cell line and animal model	Imaging	Ref.
Organic PTA	Cyanine, ICG	PDA-ICG-PEG/DOX	∼130	808nm, 0.5W/cm^2^, 20min	2.3 mg/kg(ICG),200ul	46(in mice)	4T1 tumor-bearing Balb/c mice	Magnetic Resonance, NIR thermal imaging	(26)
	Cyanine, ICG	Cal/ICG@MPs	∼380	808nm,1.5W/cm^2^, 10min	8mg/kg(ICG)	54.2(in mice)	H22/4T1 tumor-bearing mice, bilateral 4T1 orthotopic breast cancer model, 4T1 lung metastasis model	NIR thermal imaging	(27)
	Porphyrin	Chol. porph.(30 mol% cholesterol porphysomes)	∼100	670 nm, 1.9 W/cm², 60s	42 mg/kg	60	KB tumor-bearing nude mice	Fluorescence imaging, IR thermal imaging	(28)
	Semiconducting polymer nanoparticles (SPNs)	D–A semiconducting polymer/DSPE-mPEG2000/RBC membrane	<5	808 nm, 0.5 W/cm², 10 min (*in vivo*). 808 nm, 0.8 W/cm², 10 min (*in vitro*)	5 mg/kg(100 μL, 5 mg/mL)	~56 (*in vivo*), ~58 (*in vitro*, 60 μg/mL)	SMMC-7721, 4T1; BALB/c mice bearing subcutaneous 4T1 tumors	Near-Infrared Photoacoustic Imaging, fluorescence imaging, IR thermal imaging	(29)
Inorganic PTA	Noble metals nanomaterials, Gold Nanorods	Au nanorod + 5 kDa PEG-thiol coating	~13 × 47	810 nm, 2 W/cm², 5 min (*in vivo* PTT). 810 nm, 2 W/cm², 5 min (*in vitro*)	20 mg Au/kg	>70 (tumor surface)	MDA-MB-435 human breast cancer cells; nude mice bearing bilateral subcutaneous MDA-MB-435 xenograft tumors	X-ray CT, IR thermal imaging	(30)
	Carbon-based, CNT	ADP@SWNT/TNFα	1-2	808nm,1.4W/cm^2^,10s	42 mg/kg,200ul	40	HCT116 tumor-bearing Balb/c mice	NIR thermal imaging	(31)
	Carbon-based, rGO	PEG-BPEI-rGO	100-600	808nm,1W/cm^2^,5min	50 μg/mL	46.3(in mice)	RM-1 tumor-bearing Balb/c mice	NIR thermal imaging	(32)
	Transition metal, MoSe2	MoSe2@PDA	∼100	808nm,1W/cm^2^,5-10min	20 mg/kg	48-50	HeLa tumor-bearing Balb/c mice	NIR thermal imaging	(33)
	Transition metal, Iron Oxide Nanoparticles	Fe_3_O_4_ + carboxyl-terminated PEG-phospholipid	9.1 ± 1.9 (spherical)	808 nm, 2 W/cm², 10 min	10 mg/kg	~50–55	EC9706 human esophageal cancer cells; nude mice bearing subcutaneous esophageal tumor	IR thermal imaging	(34)
	Upconversion Nanoparticles (UCNPs)	NaYF_4_:Yb³^+^/Er³^+^ + SiO_2_ + Au nanorods + FA	~20.6 + SiO_2_ shell	980 nm, 1.5 W/cm², 1 min	N/A (*in vitro*)	52	OECM-1; no animal model	Upconversion Luminescence imaging, confocal imaging	(35)

#### 2.2.1 Organic PTA

Organic PTAs possess several advantages, including favorable biocompatibility and biodegradability, efficient photothermal performance, and ease of chemical modification for tumor-targeted delivery. Consequently, they have demonstrated broad potential in tumor PTT and photothermal imaging applications (36). Representative organic PTAs mainly include cyanine dyes, porphyrins, and semiconducting polymer nanoparticles (SPNs).

Indocyanine green (ICG) is the most representative cyanine-based photothermal agent. As an FDA-approved dye for clinical use, ICG has been extensively investigated due to its high NIR absorption, low toxicity, and ability to passively accumulate in tumor tissues via the enhanced permeability and retention (EPR) effect (37). However, the clinical application of free ICG is limited by several drawbacks, including rapid degradation, poor aqueous stability, low bioavailability, and nonspecific distribution (36). To overcome the poor stability and limited tumor accumulation of free ICG, different delivery systems have been developed, including inorganic nanoparticles, polymeric nanoparticles, liposomes (38), and cell membrane-camouflage nanoplatforms (26). These carriers are mainly designed to improve ICG retention in tumors, protect it from rapid clearance, and increase the efficiency of photothermal conversion. Several studies have shown the value of this strategy. Dong et al. (39) constructed a polydopamine-based nanoplatform loaded with ICG, polyethylene glycol (PEG), doxorubicin (DOX), and manganese ions (Mn). The system showed good stability and enabled imaging-guided photothermal chemotherapy in tumor-bearing mice. In another study, Li et al. (27) reported tumor cell-derived microparticles, termed Cal/ICG@MPs, with tumor-targeting ability. After laser irradiation, these microparticles generated a strong photothermal effect, induced ICD, promoted dendritic cell maturation, and activated CD8⁺ T cells. They also reduced CAFs-mediated T-cell apoptosis and improved CD8⁺ T-cell infiltration into tumors. Together, these studies suggest that ICG-based nanodelivery systems can improve both photothermal tumor killing and immune activation.

Porphyrins are also used as photothermal agents. Their earlier applications were mainly in photodynamic therapy, where they functioned as photosensitizers. This use is related to their ability to absorb light efficiently. Porphyrin-based PTT was later reported by Lovell et al. in 2011 (40). One practical advantage of porphyrins is that their molecular structure can be changed relatively easily. Functional ligands can also be introduced, while the porphyrin core generally retains good photostability (41). Their photothermal activity has been tested in animal models as well. In KB tumor-bearing mice, porphyrin treatment generated more heat and produced a stronger antitumor effect than phosphate-buffered saline (PBS) (42, 43). Free porphyrins still present several practical problems. They dissolve poorly in water, their bioavailability is limited, and only a small fraction may reach the tumor. Skin phototoxicity is another concern. For this reason, many studies use porphyrins as part of a carrier system or after chemical modification. They can instead be incorporated into nanocarriers or linked to other functional components. Lovell et al. (28) used a porphyrin-lipid system containing 30 mol% cholesterol and compared it with free porphyrin. In that study, the porphyrin-lipid formulation gave a stronger photothermal response than free porphyrin. The cholesterol-modified formulation showed a different biodistribution profile from the unmodified system. Tumor accumulation increased to approximately 3% of the injected dose per gram of tissue, while uptake by the liver and spleen was reduced. The modified system also remained in circulation for longer, with a half-life of about 8.5 h, roughly 35% longer than that of the original formulation. Semiconducting polymer nanoparticles (SPNs) are another type of organic PTA alongside cyanine dyes and porphyrins. They have been introduced into photothermal studies more recently than many conventional nanomaterials. One advantage of SPNs is that the same material can contribute to both optical imaging and heat generation. SPNs are mainly composed of -conjugated semiconducting polymers, and their absorption properties can be adjusted through changes in the conjugated backbone. Donor-acceptor polymers, for example, can be designed to absorb strongly in the NIR region. These materials have been investigated in photoacoustic imaging-guided photothermal treatment because of their photothermal conversion capacity and photostability. Their imaging and heating functions also support their use in cancer theranostic applications (29).

#### 2.2.2 Inorganic PTA

Inorganic PTAs possess several notable advantages, including deep tissue penetration capability, favorable biocompatibility, easy surface functionalization, and tunable photochemical properties (44). Major categories of inorganic PTAs include noble metal nanomaterials (45), carbon-based nanomaterials, and transition metal-based materials, all of which have been extensively investigated for PTT applications.

Noble metal nanoparticles are commonly explored in photothermal therapy because localized surface plasmon resonance (LSPR) gives them strong and tunable optical absorption (46). Gold nanoparticles (AuNPs) are one of the best studied examples (47). Their absorption can be changed by adjusting particle size and shape, while the gold surface can be functionalized with ligands or other molecules for different applications (48). These features have made AuNPs useful in many photothermal systems (49), although their behavior after systemic administration is less straightforward. Gold degrades very slowly under physiological conditions, and part of an injected AuNP dose may remain in organs such as the liver and spleen for long periods. This becomes more important when repeated dosing is considered. Particle dimensions also influence how AuNPs behave in the body. Smaller particles are often cleared more readily, whereas larger or more complex structures may provide stronger optical responses but can persist for longer periods (50). Aggregation in biological fluids is another concern because it can change both the optical properties of AuNPs and their subsequent distribution *in vivo* (51).

Several strategies have been developed to address these problems. These include using biodegradable carriers to encapsulate AuNPs, designing tumor microenvironment-responsive nanocomposites, modifying the particle surface with PEG or targeting ligands (52), and improving light delivery through fiber-optic or endoscopic systems (53). Some AuNP-based PTT platforms have already entered clinical evaluation. A representative example is AuroShell®, a gold nanoshell platform tested for prostate cancer treatment. Under near-infrared (NIR) laser irradiation, these nanoshells generate localized heat with millimeter-scale precision, while limiting damage to surrounding normal tissues. In a clinical trial involving 22 patients with prostate cancer, intravenous administration of AuroShell® at a dose of 7.5 mL/kg demonstrated favourable safety profiles without significant immune-related or systemic toxic effects. Subsequent clinical studies further confirmed the efficacy and safety of this therapeutic approach (54–56).

Beyond conventional spherical AuNPs, gold nanorods (AuNRs) have also been widely investigated as promising PTAs because of their strong and tunable NIR absorption and efficient photothermal conversion. For example, von Maltzahn et al. developed long-circulating PEG-protected AuNRs for photothermal tumor therapy. Following intravenous administration, the AuNRs accumulated in MDA-MB-435 xenograft tumors, and subsequent irradiation with an 810 nm laser (2 W/cm², 5 min) resulted in effective photothermal ablation and suppression of tumor regrowth (30). Nevertheless, concerns regarding residual CTAB-associated cytotoxicity and the limited biodegradability of AuNRs remain important challenges for their biomedical translation.

Carbon-based nanomaterials have attracted extensive interest in PTT due to their unique physicochemical properties. These materials exhibit favorable biocompatibility, tunable surface chemistry, high drug-loading capacity, and excellent photothermal conversion efficiency (57).

Carbon-based PTAs mainly include carbon nanotubes (CNTs), graphene (Gr) and its derivatives, such as graphene oxide (GO), and reduced graphene oxide (rGO), and carbon dots (CDs). To improve biocompatibility and introduce multifunctionality, carbon-based nanomaterials are usually subjected to surface functionalization (57). CNTs are highly ordered tubular carbon nanostructures that can be classified into single-walled CNTs (diameter 0.4-2.5 nm) and multi-walled CNTs (diameter ranging from several nanometers to approximately 100 nm). Owing to their exceptional electrical, thermal, and optical properties, CNTs have been widely explored in biomedical applications. However, their poor aqueous dispersibility and limited solubility restrict biological applications. These issues are commonly addressed through covalent modifications, such as esterification, or non-covalent functionalization with hydrophilic molecules such as PEG (58, 59). For example, Chen et al. (31) modified single-walled carbon nanotubes (SWNTs) with amylopectin to synthesize ADP@SWNT/TNFα complexes. This system exhibited excellent water dispersibility and plasmid DNA (pDNA) transfection capability, effectively inhibited tumor growth and metastasis, and enhanced antitumor efficacy under NIR irradiation, highlighting its considerable therapeutic potential. GO and rGO, which belong to graphene-based nanomaterials (GBNs), are two-dimensional nanostructures with remarkable physicochemical properties. The high biocompatibility and strong NIR absorption endow them with substantial potential for cancer therapy applications (60). In one study, macrophages were employed as carriers for a multifunctional system integrating DOX, rGO, and NIR-triggered drug release. Under laser irradiation, the system rapidly reached 55.8 °C within 5 min and heated tumors temperature to 46.3 °C in tumor-bearing mice, resulting in significant inhibition of tumor growth (32).

Transition metal-based nanomaterials form another group of inorganic PTAs. Metal sulfides and oxides are commonly studied because their optical, magnetic, and surface properties can be adjusted for different applications. Some of these materials absorb strongly in the NIR region, while others also provide X-ray contrast or magnetic responsiveness, which allows them to combine photothermal treatment with imaging functions (61). Molybdenum diselenide (MoSe2) is one example. Wang et al. (33) prepared a dopamine-coated MoSe2 nanocarrier (MoSe2 @PDA) with good colloidal stability and photothermal activity. In their animal study, NIR irradiation produced marked damage within the tumor and substantially reduced the number of viable tumor cells.

Metal oxides have also been explored as PTAs. Fe3O4 nanoparticles are of particular interest because they can generate heat under NIR irradiation while retaining magnetic properties. Chu et al. (34) used PEG-phospholipid-coated Fe3O4 nanoparticles in a mouse model of esophageal cancer and observed tumor growth inhibition after NIR treatment. Their magnetic behavior also makes them compatible with magnetic guidance and MRI-based imaging. The photothermal response of Fe3O4, however, varies with particle size and shape, aggregation, and surface coating, so material design has a direct effect on treatment performance.

Upconversion nanoparticles (UCNPs) are lanthanide-doped inorganic nanocrystals that can convert NIR excitation into shorter-wavelength emission. NaYF4 :Yb3+/Er3+ is a commonly used example and has been studied for optical imaging and light-responsive cancer treatment (35). UCNPs themselves usually generate little heat, so they are often coupled with a separate photothermal material when used for PTT. Chen et al. (62), for instance, combined NaYF4 :Yb3+/Er3+ nanoparticles with gold nanoparticles or gold nanorods. In this system, the UCNP component converted the incident light, whereas the gold structures provided the photothermal effect. After folic acid was introduced onto the surface, the nanocomposites showed preferential effects against OECM-1 oral cancer cells. A remaining problem is that many UCNPs have relatively low upconversion efficiency. Additional functional materials are often introduced to compensate for this weakness, but each added component also makes the system more complex.

Another noble metal nanomaterials, consisting in silver (Ag) (63), palladium (Pd) (64), and platinum (Pt) (65), exhibit tunable synthesis , favorable optical properties, and efficient photothermal performance. These materials can integrate with antibodies, peptides, polymers, chemotherapeutic agents, or immunomodulatory molecules to construct multifunctional therapeutic platforms with enhanced antitumor efficacy.

Taken together, although various PTAs possess distinct advantages, most remain at the preclinical stage of development. For many of these materials, we still need clearer data on where they go in the body, how they degrade, how long they remain, and whether they cause delayed toxicity. Also, PTT by itself rarely solves all parts of tumor treatment. Its future value will probably depend on how well it can be combined with chemotherapy, immunotherapy, or other local treatments.

## 3 From research trends to therapeutic applications: photothermal therapy combined with immunotherapy

### 3.1 Bibliometric and hotspot trend analysis of photothermal combined with immunotherapy research in recent years

To systematically evaluate the research landscape and emerging hotspots in photothermal-immunotherapy, CiteSpace software was employed to generate a keyword co-occurrence clustering map (**Figure 3**). The analysis was based on relevant publications retrieved from the Web of Science Core Collection between 2014 and 2026, including partial data from 2026 (66, 67). The resulting map identified 19 core clusters. Among them, cluster #0 (“photothermal therapy”) represented the foundational research theme, whereas cluster #8 (“cancer immunotherapy”) reflected the central direction of synergistic therapeutic development. Together, these clusters constitute the core framework of current photothermal-immunotherapy research.

**Figure 3:**
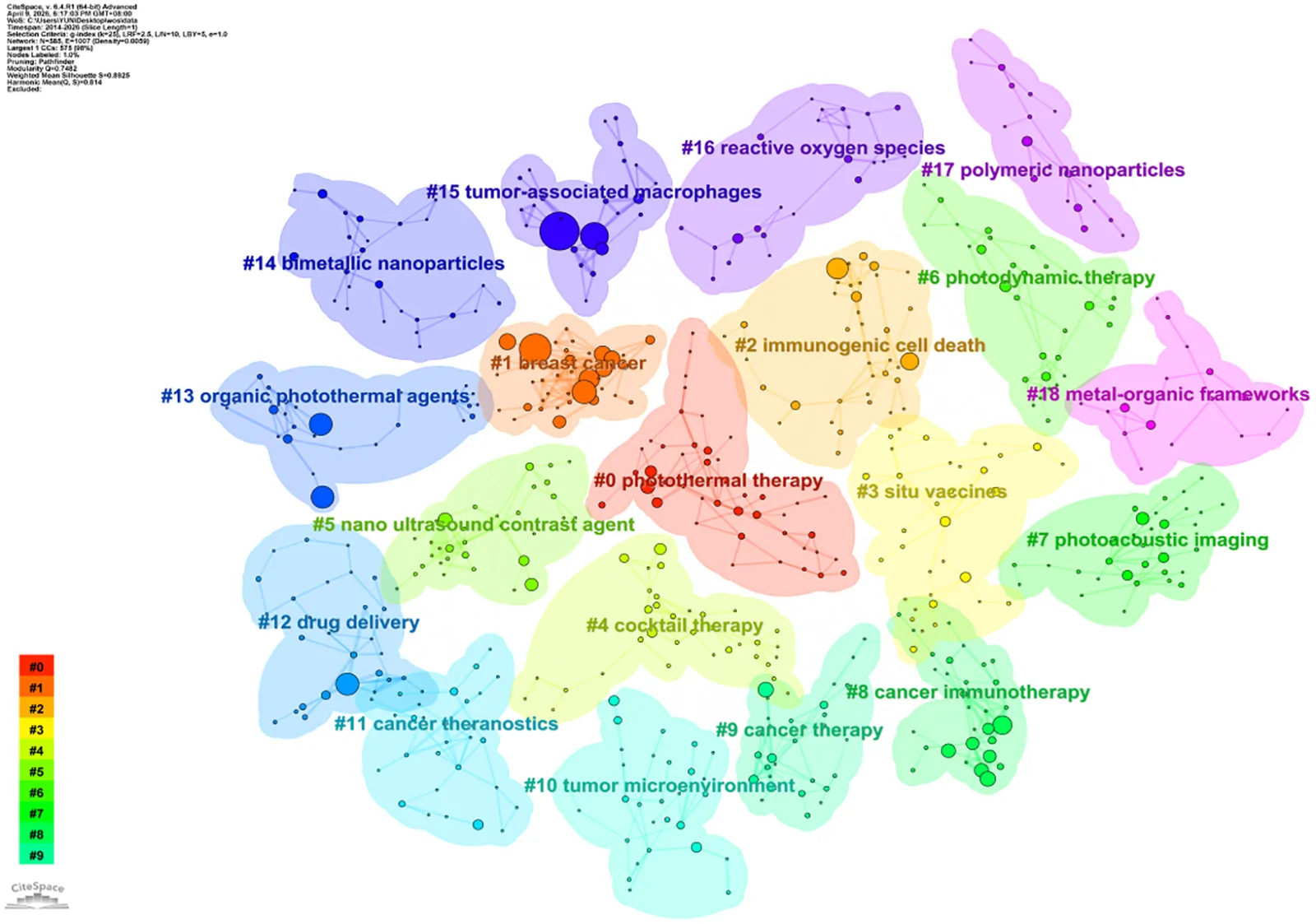
Keyword co-occurrence clustering map of literature on photothermal-immunotherapy from 2014 to 2026.

The cluster map shows that this field has developed in two main directions. One direction is the design of new PTT materials and theranostic platforms. These include organic photothermal agents (#13), bimetallic nanoparticles (#14), polymeric nanoparticles (#17), metal-organic frameworks (#18), nano-ultrasound contrast agents (#5), and photoacoustic imaging technologies (#7). On the other hand, extensive efforts have focused on immune-related mechanisms and therapeutic strategies, including immunogenic cell death (#2), tumor-associated macrophages (#15), reactive oxygen species (#16), in situ vaccines (#3), cocktail therapy (#4), and modulation of the TME (#10). The field has also started to move from basic photothermal ablation toward clinical photothermal immunotherapy, especially in specific cancers such as breast cancer.

Overall, the links among these keywords suggest a shift in the field. PTT is no longer viewed only as a local heating method. Current work is increasingly centered on immune cell death, macrophage reprogramming, in situ vaccination, nanocarrier delivery, and multimodal treatment design. These topics are likely to shape the next stage of photothermal immunotherapy research.

### 3.2 Combination of photothermal therapy and immunotherapy

PTT has been demonstrated to directly ablate tumor tissues while simultaneously enhancing tumor immunogenicity through the release of DAMPs. In addition, PTT can disrupt the dense tumor architecture and improve local blood perfusion, thereby facilitating drug accumulation within tumor tissues. PTT also promotes the recruitment and activation of endogenous immune cells, further enhancing antitumor immune responses (12). However, PTT alone is often insufficient to elicit durable and robust systemic antitumor immunity. Consequently, increasing attention has been directed toward combinational strategies integrating PTT with immunotherapy (**Table 2**). The following sections summarize the major applications and recent advances of these synergistic therapeutic approaches in cancer treatment.

**Table 2:** Research types of photothermal therapy combined with immunotherapy in the field of cancer treatment.

Research type	Photothermal agent	Immunotherapeutic agent	Brief introduction	Importance	Ref.
Photothermal therapy combined with immune checkpoint inhibitors	PCPDTBT AND AuNPs	aPD-L1	phototherapy inhibits tumor and activates immunity in CT26 tumor-bearing mice; combined with aPD-L1 antibody improves survival rate	Phototherapy combined with immune checkpoint inhibitors synergistically inhibits tumors and activates long-term anti-tumor immunity	(68)
	Au@PtNPs	PD-L1 aptamer	NTT platform integrates immunotherapy and PTT, enhances NK cell activity, and uses immune checkpoint inhibitors to kill cancer cells	Possesses potential in inducing long-term anti-tumor immune memory, providing new ideas for clinical treatment	(69)
	aPD-L1@GdCDs	aPD-L1	aPD-L1@GdCs phototherapy eradicates tumors and induces long-term immunity to prevent metastasis	Combines mild PTT with ICIs, providing a highly promising new strategy for treating immune cold tumors	(70)
	IR806	Anti-LAG3 antibody and IL-2 mRNA	Constructed an exosome drug delivery platform, combining mRNA, ICB, and PTT to reprogram the tumor microenvironment for efficient treatment of triple-negative breast cancer	Effectively reprogrammed the cold immunosuppressive TME, providing a promising strategy for the treatment of triple-negative breast cancer	(71)
	Gold nanorods	aPD-L1	A novel high-power pulsed laser device releases DAMPs to stimulate immune cells in the TME and enhance the efficacy of ICB	Pulsed PTT can effectively kill cancer cells, induce local immune activation, and enhance the efficacy of ICB therapy	(72)
	TLIF-PTT	aPD-L1, FlaB-Vax	Combined treatment of tumors with PTT, FlaB adjuvant peptide vaccine (FlaB-Vax), and anti-PD-1	Provides scientific and application basis for anti-tumor therapy combining ICD with vaccines and ICIs	(73)
	Polydopamine	aPD-L1	Constructed nanoplatform FYH-PDA-DOX to achieve multimodal imaging guidance, combining photothermal, chemotherapy, and immunotherapy to synergistically inhibit tumors and prolong survival	Developed a novel nanoplatform with imaging and synergistic therapeutic functions, providing a new strategy for the treatment of oral squamous cell carcinoma	(74)
Photothermal therapy combined with adoptive cell therapy	Sea urchin-shaped gold nanoparticles	αCD16 encoding plasmid	AuNSP@αCD16 improves TME via NIR-II PTT, mediates ADCC to activate NK cells for highly efficient tumor killing	Regarded as a highly promising candidate for future NK cell-based CAR therapies	(75)
	NPs@PBT	NKT	Local PTT treatment upregulates various chemokines and vasoactive substance C5a, followed by adoptive infusion of NKT cells	Does not rely on genetic engineering, utilizing natural or expanded NKT cells for universal or personalized therapy	(76)
	sAuNPs	CAR-T	Utilizes T cells for immune killing and nanoparticle delivery; initiates PTT for clearance when immunotherapy fails	Demonstrates a new concept of T cells as highly efficient *in vivo* nano-delivery vehicles	(77)
	PBNPs	cells expanded *in vitro* by PBNP-PTT-APC platform	Tumor cells treated with PBNP-PTT *in vitro* to induce ICD, followed by dendritic cell antigen presentation to expand tumor-specific T cells	Pioneered an antigen-independent T cell manufacturing platform, bypassing the need for known target antigens required by CAR-T or TCR-T	(78)
	PBNPs	T cells expanded *in vitro* by PBNP-PTT-APC platform	T cells expanded *in vitro* by PBNP-PTT-APC platform | PBNP-PTT induces the generation of specific T cells that can kill various tumors and secrete inflammatory factors, with significant *in vivo* efficacy	T cells from this platform significantly control glioma and prolong survival, identify key subpopulations, and possess broad applicability and clinical translation value	(79)
	PDNP	Photoporation	PDNP photoporation efficiently delivers macromolecules into T cells; 150 nm particles balance delivery efficiency and T cell function	PDNP photoporation is a promising strategy for transfecting resting T cells	(80)
Photothermal therapy combined with cancer vaccines	PDA NPs	R848	PTT-induced tumor antigens are captured by PDA NPs, while mannose targeting and R848 promote DC activation and antigen presentation.	Combines tumor ablation, antigen capture, and DC activation to enhance systemic antitumor immunity and long-term immune memory.	(81)
	PDA	Ovalbumin(OVA)	PTT directly eliminates tumor cells solely by heat; the immune response is primarily triggered by the pre-encapsulated antigen OVA of the vaccine	Achieved significant anti-tumor effects without the additional use of checkpoint blockers or immunomodulators	(82)
	MXP	Antigen OVA and CpG	MXP induces ICD via photothermal effect and serves as a nanovaccine to co-deliver OVA and CpG-ODN, realizing the synergistic effect of endogenous antigen release and exogenous immune activation	Synergistic endogenous/exogenous nanovaccine strategy	(83)
	ICG	GadoliniumTumor vessel-specific disrupting agent Vadimezan, MRI contrast Gadolinium	Enhances photothermal effect and robustly initiates the priming of tumor vaccines	Optimized delivery system significantly enhanced the photothermal ablation effect under irradiation	(84)
	PSB	mRNA vaccine	Efficiently delivers TAA encoded by WT1 mRNA to the core tumor region via light/hypoxia-driven photosynthetic bacteria PSB and 2D iMXene (Nb1.33C)	Provides a novel delivery nanocarrier for mRNA vaccines	(85)
	Mel	CpG AND c-di-GMP	Co-delivers CpG and c-di-GMP dinucleotide adjuvants, and introduces Mel as a photo-responsive component	Designed a biodegradable DNA hydrogel with immunostimulatory functions for delivering dinucleotide adjuvants and photo-responsive components	(86)
Photothermal therapy combined with cytokines	CR	IL-15	Utilizes the inherent tumor tropism of EcN to display protease-sensitive IL-15 on the EcN outer membrane via a bacterial display vector, while incorporating photothermal agent CR dye, achieving IL-15 release responsive to the TME	Provided a targeted delivery strategy for cytokines, demonstrating high TME reprogramming potential when combined with ICD inducers	(87)
	CuS	IL-12	Constructed a CuS-loaded CSP nano-delivery system, achieving the combination of NIR-II photothermal therapy and *in situ* IL-12 gene immunotherapy	First attempt to combine NIR-II PTT and IL-12 cytokine gene therapy, achieving significant systemic therapeutic effects via *in situ* administration	(88)
	IR806	IL2 mRNA, anti-LAG3 antibody	Constructed exosome-camouflaged lipid nanoparticles ALEvs@LNPs for co-delivery of photothermal agent IR806, IL2 mRNA, and anti-LAG3 antibody	Proposed a new strategy combining cytokine-mediated mild PTT, ICB, and IL2	(89)
	Conjugated polymer	IL-12	Constructed an NIR-II light-responsive conjugated polymer nanoparticle system, achieving precise *in situ* release of IL-2 under mild photothermal conditions	First achievement of customized release of IL-2 from 3D tumor spheroids to enhance immunotherapeutic efficacy	(90)
	KRN-7000 photochromic derivative	Cytokines	Precisely regulates NKT cell activation and cytokine release via illumination	Selective activation of cytokines in the tumor microenvironment with high spatiotemporal precision	(91)
Photothermal therapy combined with oncolytic virus therapy	ICG	OVV	Constructed indocyanine green-loaded and platelet membrane-encapsulated oncolytic vaccinia virus system PIOVV	Advances phototherapy, oncolytic virology, drug delivery, and tumor-specific targeting	(92)
	Carbon nanotubes	VSV	Established a synergistic therapeutic system combining VSV oncolytic therapy with carbon nanotube-mediated photothermal therapy	First study combining VSV and carbon nanotubes for treatment	(93)
	IR780	oAd	Intranasal pH-sensitive oAd/IR780 system for glioblastoma therapy.	Enables non-invasive brain delivery and synergistic oncolytic-photothermal therapy.	(94)
	IR1061	OVV	SFOVV@IR1061 for NIR-II imaging-guided photothermal-oncolytic therapy of DLBCL.	Enhances tumor targeting, immune activation, and synergistic photothermal-oncolytic therapy.	(95)
Photothermal therapy combined with bispecific antibodies	ICG	Nb-MuTE	Constructed a nanobody-guided multifunctional T cell engager Nb-MuTE, capable of targeting tumor and T cells combined with ICG photothermal effect	Integrates PTT with T cell immunotherapy via Nb-MuTE	(96)
	HPDA	MK-8628	Constructed hollow polydopamine nanoplatform HPDA@MK loaded with MK-8628	Achieved synergy between photothermal therapy and c-MYC/PD-L1/CD47-targeted immunotherapy	(97)
	HMRu	SS-Fc	Constructed hollow mesoporous ruthenium nanosystem HMRu@RBT-SS-Fc loaded with fluorescent anti-tumor complex RBT and modified with bispecific antibody SS-Fc	Bispecific antibody-targeted drug delivery and NK cells integrated with photothermal therapy, providing a novel platform for image-guided precise combination anti-cancer therapy	(98)

#### 3.2.1 Combination of photothermal therapy and immune checkpoint inhibitors

Immune checkpoint inhibitors (ICIs), including drugs directed against PD-1/PD-L1 and CTLA-4, are used to remove inhibitory signals that limit T-cell activity. They tend to work better when antitumor immune cells are already present in the tumor or can be recruited during treatment. This is not always the case in solid tumors. Some tumors release little antigen, contain few infiltrating T cells, or maintain a local environment that suppresses immune activity, all of which can reduce the effect of checkpoint blockade.

PTT provides a potential way to improve this setting. PTT promotes the release of tumor-associated antigens and danger signals by inducing local tumor damage, especially when ICD is triggered. These may enhance antigen performance and increase T-cell priming. ICIs can then act on this newly activated immune response by blocking checkpoint-mediated T-cell exhaustion or immune escape. Therefore, the biological rationale for combining PTT with ICIs is not simply additive tumor killing, but the coupling of antigen release, immune activation, and checkpoint blockade.

The efficacy of this combination is strongly affected by treatment conditions. The magnitude and quality of ICD depend on irradiation dose, temperature, exposure time, and tumor type. Excessive heating may cause tissue destruction and nonspecific inflammation, whereas insufficient heating may fail to generate enough antigenic and danger signals. In addition, PTT-induced inflammation and antigen release must be temporally matched with ICI activity. Otherwise, the immune response may remain weak or be counterbalanced by compensatory immunosuppressive pathways. Several nanoplatforms have been designed for this purpose. Instead of acting only as heat generators, they are often built to combine PTT with immune regulation, targeted delivery, or other treatment functions. Lee et al. (68) reported CPN4, which combined photothermal and photodynamic effects and showed improved antitumor activity (**Figure 4A**). Chang et al. (69) tested NTT-1 in a bilateral Lewis lung carcinoma model in C57BL/6J mice. Mild PTT induced ICD, and anti-aPD-L1 treatment blocked PD-L1-mediated immune escape. Together, the two treatments caused complete tumor regression, extended survival, and generated durable immune memory. This result supports the idea that well-controlled PTT may turn local tumor damage into a broader systemic antitumor response. However, the immunological evaluation in this study remained relatively limited. ICIs were only administered via intraperitoneal injection rather than co-delivered within the nanoplatform, and immune analysis primarily focused on CD8/T cell/regulatory T-cell (Treg) and M1/M2 macrophage ratios, without more comprehensive characterization such as cytokine profiling, single-cell RNA sequencing (scRNA-seq), or detailed immune subset analysis. Fan et al. (70) further developed an all-in-one therapeutic nanoplatform (aPD-L1@GdCDs combined with NIR irradiation), which significantly enhanced CD8+ T cell infiltration, reduced tumor burden, and promoted the conversion of immunologically "cold" tumors into "hot" tumors (**Figure 4B**). Nevertheless, this study also presented several limitations. The use of a 650 nm laser provided relatively limited tissue penetration compared with conventional 808 nm NIR irradiation. In addition, the study was constrained by a relatively small sample size and lacked a detailed quantitative analysis of the synergistic contributions of each therapeutic component.

**Figure 4:**
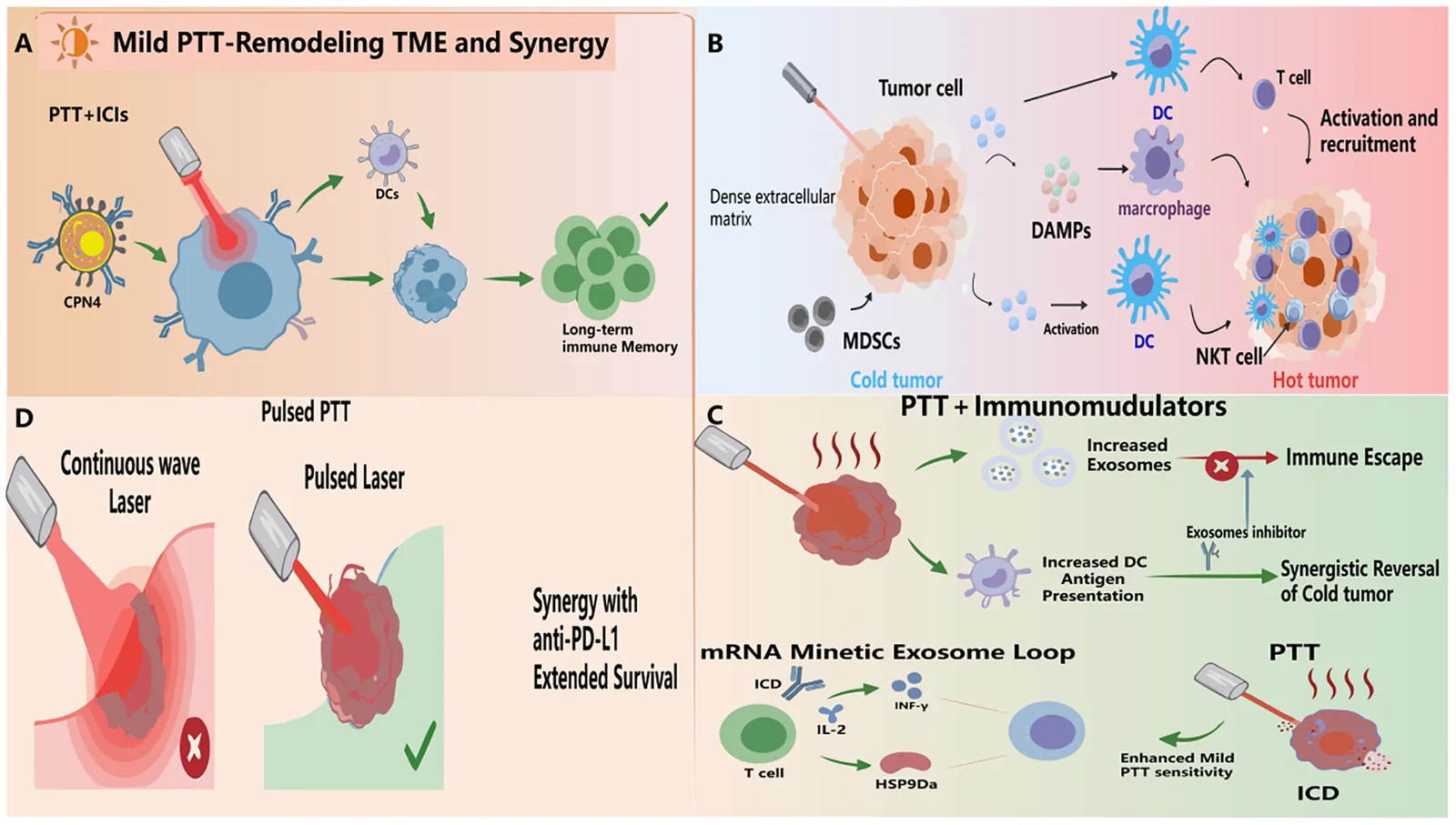
Schematic illustration of synergistic effects of PTT and ICIs. **(A)** CPN4-mediated PTT/PDT combined with anti-PD-L1. This combinatorial strategy achieves complete tumor regression, improved survival and long-term immune memory *in vivo*. **(B)** The aPD-L1@GdCDs nanoplatform mediates mild PTT (45–50 °C) to trigger ICD and antigen release. Liberated aPD-L1 blocks immune escape, facilitates CD8^+^ T-cell infiltration and transforms cold tumors into hot tumors. **(C)** Mild-PTT-centered multimodal synergistic network. Mild PTT cooperates with exosome inhibition, PD-L1 blockade and mRNA-derived IL-2-driven T-cell expansion. IFN-γ-mediated HSP90α down-regulation enhances PTT sensitivity to construct a positive feedback loop. **(D)** Pulsed PTT, relative to continuous-wave laser, alleviates thermal damage to healthy tissues and exerts potent anti-tumor effects when combined with anti-PD-L1.

Current work in this area has moved beyond simple PTT plus antibody treatment. Many studies now focus on nanoplatforms that combine heat generation, immune activation, and drug delivery in one system. These platforms are designed to improve the efficacy of PTT combined with ICIs. Ideally, they should induce tumor ablation, trigger ICD, and reshape the TME at the same time. Some systems can also control local temperature more precisely, which may help favor ICD over non-immunogenic necrosis. This point still needs caution. Not all tumors respond to PTT in the same way, and not every PTT setting can induce meaningfu ICD. In some studies (68–70), the evidence remains mainly correlative. The work shows immune changes after treatment, but does not fully prove the causal link between PTT-induced ICD and systemic antitumor immunity. As a result, there is still no complete translational framework that connects nanoplatform design, immune mechanism, treatment schedule, safety evaluation, and clinical outcome. Another issue is manufacturing. The main bottleneck is no longer only whether a multifunctional nanoplatform can be made. The harder question is whether it can be produced reproducibly and safely under Good Laboratory Practice (GLP) and Good Manufacturing Practice (GMP) standards. Many systems are structurally complex and difficult to scale up. This helps explain why most studies remain in cell or animal models. Clinical translation slowed by safety concerns, especially the accumulation in the body or cause of unexpected toxicity wihthin nanohybrids. Only a limited number of clinical trials have been initiated, and some candidates have failed because of safety or adverse effects. To overcome the therapeutic resistance of immunologically cold tumors, various strategies have been developed to achieve deeply remodeling of the TME. Exosomes are known to play a key role in tumor immune escape, and elevated circulating exosomal PD-L1 levels have been associated with reduced responsiveness to ICIs (99). Therefore, combining PTT and ICIs with exosome inhibition represents a promising strategy for converting cold tumors into immunologically hot tumors. Additionally, genetically engineered tumor cell-derived exosome coatings can achieve efficient drug encapsulation and targeted delivery while simultaneously conferring homologous tumor-targeting and immune-evasion properties to nanoparticles. For example, Chen et al. (89) validated in a breast cancer mouse model that combining mild PTT with exosome inhibition effectively remodeled the immunosuppressive TME. Their study observed that while mild PTT increased exosome secretion, which could potentially promote immune escape, it also upregulated antigen-presenting markers and immune-related genes in DCs, thereby improving the efficacy of ICB (**Figure 4C**). Consequently, the addition of exosome inhibition further improved TME remodeling and promoted the transition from cold to hot tumors. The integration of messenger RNA (mRNA) therapy with PTT and ICIs has also shown promise for enhancing antitumor immunity in cold tumors. Sun et al. (71) developed a biomimetic exosome-based platform integrating mRNA therapy, ICIs, and mild PTT. Importantly, these therapeutic modalities did not function independently but instead formed a synergistic positive feedback loop.

ICB and IL-2 can both enhance T-cell activity, with IFN-γ being one of the cytokines released during this response. In some experimental models, higher IFN-γ signaling has been linked to lower HSP90α levels in tumor cells, which may make them more responsive to mild PTT. Mild PTT can also induce immunogenic cell death and promote the release of tumor antigens. This may improve tumor immunogenicity and support subsequent T-cell responses. Sustained local IL-2 expression provides an additional stimulus for T-cell expansion and activation, which may help maintain the immune response during combination treatment.

The way heat is delivered is therefore important. Pulsed irradiation has been explored as an alternative to continuous-wave heating because it may reduce damage to surrounding tissue while maintaining the photothermal effect. Farivar et al. (72) tested this approach in a subcutaneous MB49 bladder cancer model in C57BL/6 mice and examined how pulsed PTT influenced ICD-related responses and tumor control. Pulsed treatment caused less damage to nearby normal tissue than continuous-wave PTT. When combined with anti-PD-L1 therapy, it also slowed tumor growth and prolonged survival. The study, however, did not include a detailed analysis of the immune response. Tumor-infiltrating lymphocytes, cytokine profiles, flow-cytometric characterization of T-cell populations, and single-cell RNA sequencing were not examined, leaving the immune mechanism of the combination only partly defined.

Similarly, Thiruppathi et al. (73) established a bilateral DD-Her2/neu breast cancer model in BALB/c mice to investigate the abscopal effect and inhibition of metastasis, thereby demonstrating the induction of systemic antitumor immunity. They further employed an orthotopic large-tumor model to mimic advanced bulky tumors in clinical settings. Their finding showed that PTT combined with a therapeutic vaccine still resulted in tumor recurrence, whereas the additional incorporation of anti-PD-1 therapy achieved complete tumor eradication and durable recurrence-free survival. Nevertheless, ICIs in this study were still administered systemically rather than integrated into the nanoplatform, and the immune characterization lacked single-cell-level mechanistic analysis. Multimodal combination therapies have demonstrated substantial potential for achieving synergistic antitumor effects. In particular, the integration of PTT, chemotherapy, and ICIs can simultaneously activate antitumor immunity while suppressing treatment-induced PD-L1 upregulation. Gu et al. (74) used a homologous oral squamous cell carcinoma mouse model and found that although the combination of PTT and chemotherapy increased PD-L1 expression, the addition of aPD-L1 antibodies effectively blocked this immunosuppressive pathway. This strategy remodeled the TME, further enhanced T-cell-mediated cytotoxicity, and ultimately achieved superior synergistic photothermal-chemo-immunotherapeutic efficacy.

Whether through the incorporation of mRNA therapy or exosome inhibitors into the PTT-ICI framework, these approaches are fundamentally based on a synergistic “multiplicative” therapeutic rationale rather than a simple additive effect. However, from the perspective of mechanistic discovery and validation, most studies (72–74) largely focused on conventional apoptotic pathways. For instance, analyses involving reactive oxygen species (ROS) generation, cell cycle arrest, and flow cytometric apoptosis assays can partially explain how combination therapies promote ICD. Nevertheless, these studies rarely investigate whether other forms of regulated cell death, beyond apoptosis, are involved in the therapeutic process. This limitation is common among current reports and may partly reflect the primary focus of these studies.

From the standpoint of material engineering and theranostics integration, some studies have achieved multimodal imaging and self-monitoring functions for guided drug release (72). However, as noted previously, pulsed PTT systems still lack real-time thermal monitoring and automated feedback regulation. Without the ability to continuously monitor tissue temperature and automatically adjust laser power output, the risk of thermal injury remains a major obstacle to clinical translation. Furthermore, the therapeutic efficacy of PTT combined with ICIs is highly dependent on treatment parameters, including dosing sequence, administration intervals, and dosage combinations. To date, standardized therapeutic protocols have not yet been established.

Some studies have reported strong abscopal effects after combination therapy (73). However, this effect is not consistent and is still hard to predict. Improving the stability and reproducibility of systemic antitumor responses therefore remains an important goal. Another issue is that many studies only assess the overall tumor response, without examining how different regions within the same tumor respond to treatment. This may lead to an incomplete understanding of therapeutic efficacy. Intratumoral injection is also used in some studies (68, 72, 73), but this route limits clinical use. Nanoparticle-based co-delivery systems may help improve tumor accumulation and reduce systemic toxicity. Even so, the field still lacks large clinical studies and long-term safety data, especially on immune-related adverse events. Some newer nanoplatforms can combine therapeutic and diagnostic functions in one system (74). These designs are promising, but translation remains difficult. One major barrier is the difference between mouse models and the human immune system. More clinically relevant humanized models are therefore needed to support the future development of photothermal immunotherapy.

#### 3.2.2 Photothermal therapy combined with adoptive cell therapy

Most current studies employ PTT prior to adoptive cell therapy (ACT), using photothermal treatment to partially reduce tumor burden, remodel the TME, and establish chemokine gradients that facilitate subsequent adoptive immune cell infiltration and activation. Murciano et al. (100) found that the immunosuppressive TME restricts T cell filtration, reduces the activity of tumor-infiltrating lymphocytes (TILs), and downregulates immune checkpoint molecule expression. Consequently, current strategies combining PTT with ACT primarily rely on the TME-remodeling capacity of PTT to establish a microenvironment more favorable for immune cell function.

The combination of TME-modulating PTT and ACT mainly aims to overcome two major limitations of ACT monotherapy: the physical barriers that impede immune cell infiltration and the insufficient recognition signals that result in ineffective immune activation. For example, in the treatment of solid tumors such as non-small cell lung cancer requiring real-time therapeutic monitoring, Lin et al. (75) developed an AuNSP@αCD16 platform combined with PTT. This system disrupted tumor physical barriers and upregulated Heat Shock Protein 70 (HSP70) expression while simultaneously targeting αCD16 on the tumor surface. Importantly, the platform delivered genes directly to tumor cells rather than immune cells, thereby achieving localized gene transfection while minimizing systemic toxicity (**Figure 5A**). This strategy subsequently activates a multilevel immune response involving NKT, NK, and CD8+ T cells, ultimately establishing durable immune memory. *In vivo* experiments demonstrated that the combined AuNSP@αCD16 + PTT + NK-cell therapy achieved complete tumor regression.

**Figure 5:**
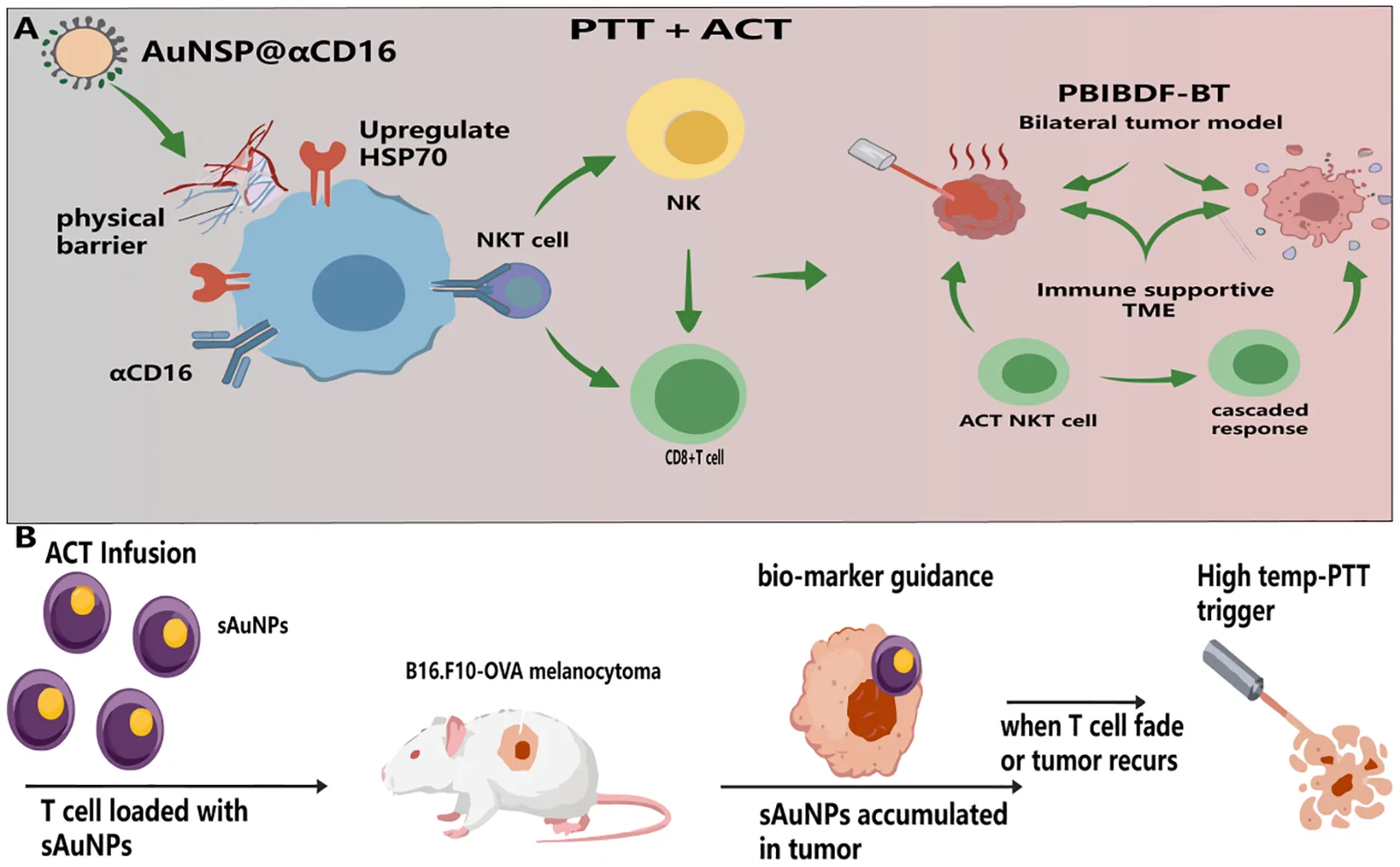
Schematic illustration of the synergistic antitumor effects of PTT combined with ACT. **(A)** AuNSP@αCD16-based PTT disrupts tumor physical barriers and elevates HSP70. αCD16 enables tumor-selective gene transfection avoiding systemic toxicity, activating NKT, NK, CD8^+^ T-cell responses and long-term immune memory. Combined AuNSP@αCD16/PTT/NK-cell therapy achieves complete tumor regression *in vivo*. **(B)** For B16.F10-OVA melanoma-bearing mice, sAuNP-carrying T-cells are administrated for ACT. Tumor-accumulated sAuNPs and T-cell infiltration guide treatment timing. High-temperature PTT is applied upon T-cell exhaustion and tumor recurrence. This sequential regimen inhibits tumor growth, improves survival with minimal systemic toxicity.

Similarly, Li et al. (76) utilized the conjugated polymer PBIBDF-BT as a photothermal agent in a bilateral B16F10 melanoma mouse model. PTT was selectively applied to only one tumor lesion to induce the formation of an immune-supportive TME, followed by the adoptive NKT cell transfer. The results showed that PTT promoted the recruitment and activation of NKT cells and initiated downstream immune cascades. However, these findings were not further validated in additional tumor models, limiting the generalizability of the conclusions. In contrast to TME-remodeling mild PTT strategies, the combination of high-temperature ablative PTT with ACT adopts a different therapeutic sequence and mechanism. Rather than administering PTT before ACT, Kim et al. (77) first performed ACT in C57BL/6 mice bearing subcutaneous B16.F10-OVA melanoma tumors. By internally loading T cells with small AuNPs, they used the intratumoral accumulation of small AuNPs and T cell infiltration as biomarkers to guide subsequent treatment timing. When T cell function weakened and tumor relapse occurred, high-temperature PTT was triggered to achieve rapid tumor ablation. This strategy significantly inhibited tumor progression, prolonged survival, and produced no obvious systemic toxicity(**Figure 5B**). Notably, Kim’s study demonstrated the feasibility of using immune cells as delivery vehicles to enhance nanoparticle accumulation within tumor. However, the study did not comprehensively evaluate the potential collateral damage to surrounding healthy tissues caused by high-temperature thermal ablation. Compared with mild PTT and photoporation-based approaches, the immunotherapy first, high-temperature PTT-second strategy offers several advantages, such as reduced risk of therapeutic resistance, rapid tumor debulking, and strong local tumor eradication capacity. Nevertheless, the possibility of severe damage to adjacent normal tissues remains a major limitation and a significant obstacle to clinical translation.

Combining PTT with CAR-T therapy is mainly used to deal with antigen heterogeneity in solid tumors. This is a major reason why CAR-T cells directed at a single antigen often show weak effects in these tumors. Sweeney et al. (78, 79) developed a Prussian blue nanoparticle-based PTT platform for heterogeneous solid tumors, including glioblastoma. In their design, PBNP-PTT was first used to damage tumor cells and induce ICD. This released a broad set of tumor antigens. Dendritic cells then processed these antigens and presented them to T cells, leading to the expansion of tumor-specific T cells. In an orthotopic GBM xenograft model, this approach reduced tumor growth and improved survival. In a later study, PBNP-PTT was used *in vitro* to prepare tumor antigens for the generating multifunctional tumor-specific T cells for ACT. These T cells recognized tumor targets, secreted IFN-gamma and TNF-alpha, and showed higher CD107a expression, suggesting increased killing activity. In orthotopic GBM xenograft models, the transferred T cells slowed tumor progression and improved survival (78). Berdecka et al. (80) focused on T-cell delivery, another important issue in ACT. By adjusting PDNP size, they achieved about 60% delivery efficiency in unstimulated human T cells, while largely preserving cell growth, activation marker expression, and cytokine release. This strategy may provide a non-viral route for CAR-T-related engineering. It could reduce cost and some safety concerns linked to viral vectors. It also avoids long pre-activation or extended cell culture, which may be useful for faster ex vivo T-cell preparation. Li et al. (76) reported another related platform, named CISP, for highly metastatic tumors such as melanoma. This system used quercetin-iron nanoparticles as the photothermal component, carried the STING agonist SR-717, and was coated with beta-cyclodextrin-treated PD-1-overexpressing T-cell membranes. The design improved tumor targeting and combination efficacy. Still, the long-term fate of the low-cholesterol T-cell membrane coating was not fully studied, including its stability, degradation, and possible metabolic products.

Current platforms have shown antitumor effects and acceptable short-term safety in preclinical models. However, several issues remain unresolved. Temperature control is especially important. Mild PTT may be better for immune activation because it can induce ICD and alter the TME without causing extensive necrosis. This makes it more suitable for approaches that try to trigger systemic immunity or combine PTT with ACT (75, 76). High-temperature PTT, by contrast, can rapidly reduce local tumor mass. It may be useful for large tumors or for tumors that respond poorly to immunotherapy (77). The drawback is that strong local inflammation and tissue injury may impair the survival, infiltration, or function of transferred immune cells. Treatment timing is another issue. The best order of PTT and ACT probably depends on the mechanism of each combination and needs direct experimental testing. At present, few studies have compared mild and high-temperature PTT in ACT-based combinations. From a safety point of view, low-dose or mild PTT may be a better starting condition because it can promote ICD while reducing unnecessary tissue damage.

However, ACT itself can induce severe immune-related adverse events, including neurotoxicity and cytokine release syndrome (CRS) (101). These toxicities are primarily associated with widespread endothelial activation and dysfunction triggered by systemic cytokine storms rather than direct neuronal damage caused by CAR-T cells. Local tissue damage from PTT can trigger inflammation and activate the coagulation cascade. Similarly, the activation of immune effector cells during ACT often leads to CRS and downstream coagulation disorders. When used together, these treatments risk cross-amplifying toxicity along the inflammation-coagulation axis. For example, Sweeney’s study (78) demonstrated that tumor cells treated with PBNP-PTT release high levels of inflammatory cytokines, including IL-6, TNF-α, and IFN-γ, all of which are known mediators of endothelial activation and blood-brain barrier disruption (79, 101). Nevertheless, the actual *in vivo* activation status and systemic immune effects of PBNP-PTT- generated T cells remains insufficiently characterized. However, PTT does not simply drive inflammation in a single direction. Its actual impact on ACT-induced immunotoxicity depends heavily on the temperature used, the specific treatment modality, the tumor type, and the timing of the intervention. Because these factors vary so much, a predictable additive toxic signal rarely emerges. Still, when combined with PTT-induced inflammatory responses, such immune-related toxicities may potentially be exacerbated. Clinically, this might show up as glaring coagulation abnormalities, higher chances of bleeding or thrombosis, and more severe CRS complications (102, 103).

So far, reports on these combined toxicities remain scarce. One major reason is that directly integrating PTT with ACT is still rare in routine clinical practice (104). Short-term follow-up studies also tend to miss these delayed events. These findings suggests that future studies combining PTT with CAR-T therapy could benefit from more comprehensive monitoring of endothelial activation biomarkers, such as the angiopoietin-2/angiopoietin-1 (Ang-2/Ang-1) ratio and von Willebrand factor (vWF), to predict the risk of severe neurotoxicity and systemic inflammatory complications, besides, future trials need to extend their observation windows. Tracking patient cytokine profiles over longer periods will be essential to catch any delayed toxicities that would otherwise go unreported. In addition, concerns regarding the potential cytotoxicity and long-term biosafety of nanocarriers have driven the development of biodegradable organic nanomaterials, which may further facilitate the clinical translation of PTT combined with ACT.

#### 3.2.3 Photothermal therapy combined with cancer vaccines

Cancer vaccines are used to stimulate immune responses against tumor-associated antigens (105). They include prophylactic vaccines, such as the human papillomavirus vaccine, and therapeutic vaccines used after tumor development. Therapeutic vaccines are expected to activate antitumor immunity, remove existing tumor cells, and form immune memory that may reduce recurrence. Sipuleucel-T is a typical example and was the first dendritic cell-based cancer vaccine approved by the U.S. FDA (106, 107). Some clinical studies have shown that cancer vaccines can benefit patients, including reducing tumor burden and improving survival in certain cases (108). Even so, vaccines used alone often show limited efficacy. This is related to weak antigen expression, antigen heterogeneity, and the suppressive tumor microenvironment. PTT has therefore been introduced as a partner for cancer vaccines. One common strategy is to use PTT to release tumor antigens in situ. Local heating damages tumor cells and releases endogenous antigens. These antigens can then support vaccine-driven immune activation. This approach is useful when the vaccine itself cannot induce strong ICD. Li et al. (81) designed an in situ nanovaccine using polydopamine nanoparticles coated with acid-responsive liposomes and functionalized with mannose, with R848 incorporated as an immune adjuvant. Following NIR irradiation, the polydopamine core generated a photothermal effect that damaged tumor cells and promoted the release of tumor-associated antigens. These antigens could then be adsorbed onto the nanoparticles, while the presence of R848 enhanced dendritic-cell activation and facilitated subsequent antigen presentation. These nanoparticles then moved to tumor-draining lymph nodes, where they promoted dendritic cell maturation and antigen presentation. R848 also activated TLR7/8 signaling and increased pro-inflammatory cytokine production. Together, these effects enhanced antigen-specific antitumor immunity and produced a strong photothermal-immunotherapy response.

The second strategy combines PTT-induced tumor ablation and vaccine- encoded antigens. In this mode, the primary role of PTT is local tumor eradication through thermal ablation, whereas the antitumor immune response is mainly elicited by predefined antigens incorporated into the vaccine formulation. A representative example is the ovalbumin (OVA)-based nanovaccine MSNs-ABC@PDA-OVA (82). OVA is frequently employed as a model antigen because of its availability, low cost, and strong immunogenicity. This nanovaccine achieved an OVA loading efficiency of up to 92.2%. Under 808 nm laser irradiation, the PDA component directly ablates B16-OVA melanoma cells through PTT. Simultaneously, the acidic TME promotes the decomposition of ammonium bicarbonate, accelerating OVA release and enhancing antigen cross-presentation. *In vitro* studies demonstrated significantly improved antigen release efficiency and increased CD4^+^/CD8^+^T cell responses. In a B16-OVA melanoma mouse model, the combination of nanovaccine administration and laser irradiation not only markedly suppressed primary tumor growth but also achieved a 75% rate and effectively prevented tumor recurrence. Beyond these two approaches, some studies have tried to combine endogenous and exogenous vaccination in one system. Zhang et al. (83) developed a Ti3C2 MXene-based nanoplatform called MXP. This platform acted both as an in situ vaccine and as an exogenous nanovaccine. Its photothermal effect induce ICD in tumor cells and released endogenous tumor antigens and danger signals. These signals helped dendritic cells mature and present antigens. At the same time, the MXP@OC formulation delivered the model antigen OVA together with CpG-ODN, an immune adjuvant. This further activated dendritic cells and promoted adaptive immune responses. Through this dual-vaccine strategy, the MXene platform suppressed tumor growth and enhanced systemic antitumor immunity.

PTT-based *in situ* vaccination can still be limited by residual tumor cells and an immunosuppressive TME. Poor accumulation of photothermal agents in tumor tissue is another problem, since it can weaken local heating. Zhao et al. (84) tried to address this by developing multifunctional nanoparticles named VIGd-NPs. These particles were formed by chemical self-assembly of vadimezan, ICG, and Gd. Vadimezan disrupts tumor blood vessels and has entered phase III clinical trials. With Gd-enhanced MRI guidance, the system improved intratumoral delivery of ICG and Gd. This led to stronger tumor ablation and partly relieved the immunosuppressive TME. As a result, both imaging and *in situ* vaccine efficacy were improved.

Therapeutic cancer vaccines include mRNA vaccines, dendritic cell vaccines, peptide vaccines, DNA vaccines, and oncolytic virus vaccines (109). Among them, mRNA vaccines have attracted particular interest because of they are easy to design and can be produced quickly. However, their clinical application still faces several challenges, including biological instability, large molecular size, insufficient *in vivo* delivery, and the need to achieve an appropriate balance between beneficial innate immune activation and excessive inflammatory responses. Moderate innate immune sensing of mRNA can provide a desirable self-adjuvanting effect and enhance antitumor immune responses, whereas excessive activation may lead to inflammatory toxicity and limit the tolerable dose. To address these limitations, Zhang et al. (85) constructed a novel nano-delivery system, PSB@Nb1.3C/mRNA, which integrates photosynthetic bacteria (PSB) and two-dimensional iMXene (Nb1.3C) to facilitate the efficient delivery of Wilms' tumor 1 (WT1) mRNA-encoded tumor-associated antigens to the tumor core. Guided by light-responsive and hypoxia-targeting properties, both PSB and Nb₁.₃C contribute to photothermal conversion and induce ICD. This process promotes the release, expression, and presentation of WT1 antigens, enhancing antitumor immune responses. *In vivo* experiments showed that this strategy reduced distant tumor growth and postoperative lung metastasis. DNA-based vaccine systems have also been combined with PTT. Wu et al. (86) prepared a CpG-containing DNA hydrogel by rolling-circle amplification, incorporated c-di-GMP, and then coated the construct with melanin to obtain Mel/G/DH. Under 808 nm NIR irradiation, the melanin layer produced local heating. In CT26 cells, Mel/G/DH promoted calreticulin exposure and enhanced dendritic-cell maturation. When administered locally in mice, the system eliminated the primary tumor after photothermal treatment and increased DC activation in draining lymph nodes. Distant tumor growth was also inhibited. The treated mice showed 100% survival, compared with a much lower survival rate in the Mel/G+NIR control group. Analysis of distant tumors further showed increased cytotoxic T-cell infiltration together with reduced Treg abundance. These findings suggest that the platform did more than deliver a DNA vaccine locally; it also helped generate a broader antitumor immune response.

Other vaccine formats have also been paired with PTT, including bacterial outer-membrane-vesicle vaccines (110), tumor-cell-membrane-coated nanovaccines (111), and exosome-based systems (112). The rationale for these combinations is that photothermal treatment changes more than tumor temperature. Tumor cells damaged by heat release endogenous antigens and other intracellular signals, which can provide additional material for immune recognition. This may be useful in tumors with substantial antigen heterogeneity, where a predefined vaccine antigen set may capture only part of the tumor repertoire. PTT can also promote dendritic-cell activation and antigen presentation. At the same time, local heating may alter the tumor microenvironment and, in some settings, make immune-cell entry into the tumor easier (108). In this way, PTT can contribute both antigenic material and local immune modulation, which helps explain why it has been combined with cancer vaccines. A fundamental issue lies in the difficulty of precisely controlling the dual immunological effect of PTT. On the one hand, high-temperature ablative PTT can rapidly reduce tumor burden; on the other hand, excessive thermal damage may denature released antigens and induce fibrotic tissue formation, potentially impairing immune cell infiltration and limiting subsequent vaccine efficacy. For example, studies based on OVA vaccine models (82) suggest that excessive thermal ablation may compromise the immunological benefits of antigen release. Conversely, mild PTT is generally more favorable for inducing ICD and initiating antitumor immunity. However, its therapeutic effectiveness relies on highly precise thermal dose control and may vary substantially across different tumor types due to tumor heterogeneity and differences in ICD induction thresholds. Another major challenge involves the efficient capture, delivery, and presentation of tumor antigens. Although PTT can effectively release endogenous antigens, strategies that ensure their efficient capture by nanovaccines and subsequent transport to tumor-draining lymph nodes remain limited.

Several nanoplatform designs have been reported to address this issue (83). However, quantitative data are still limited regarding *in vivo* antigen capture, lymph node targeting, and competition with endogenous antigen-processing pathways. Similarly, nucleic acid delivery systems using mRNA- or DNA (85, 86) face practical issues, including systemic degradation, cytosolic entry, and efficient antigen expression. Translational application introduces additional practical constraints. Patient-specific variability in tumor antigens makes it difficult to design standardized formulations. Meanwhile, nanocarriers require further evaluation in terms of biocompatibility, degradation, and large-scale manufacturing. In photothermal systems, limited tissue penetration depth also restricts treatment efficacy in deep-seated tumors.

#### 3.2.4 Photothermal therapy combined with cytokines

Cytokines are small signaling proteins produced by immune cells, stromal cells, and tumor cells. They regulate immune cell differentiation, proliferation, and activation, and they also shape the TME. Many cytokines are involved in antitumor immunity, but their expression and function are often disturbed during tumor progression. Their clinical use is also limited by short half-life and dose-related toxicity (113). For this reason, cytokine therapy has been combined with PTT to link local tumor destruction with systemic immune activation. Wang et al. (87) developed a biohybrid bacterial system based on probiotic E. coli Nissle 1917 (EcN) for the combined delivery of PTT and IL-15. This system used the natural tumor-targeting ability of EcN. Protease-sensitive IL-15 was displayed on the bacterial outer membrane, and Congo red (CR) was added as the photothermal agent. After NIR irradiation, CR produced mild local heating. This induced ICD and promoted the release of DAMPs and TAAs. At the same time, EcN accumulated in the tumor and delivered IL-15 locally. Local IL-15 then supported the expansion of NK cells and CD8+ T cells, while PTT improved immune cell infiltration within the tumor. Furthermore, the addition of anti-PD-1 therapy alleviates immunosuppression, significantly improves therapeutic efficacy against both cold and hot tumors. This approach not only reduces the systemic toxicity associated with IL-15 administration but also overcomes the challenge of limited tissue penetration in solid tumors. To solve the short half-life and off-target toxicity of cytokines, strategies beyond tumor-targeted cytokine delivery have also been explored. 

One approach is to produce cytokines directly within the tumor rather than deliver them systemically. Lin et al. (88) developed CSP@IL-12, a nanoparticle platform that combines NIR-II photothermal treatment with local IL-12 expression. After irradiation, the system generated heat at the tumor site while supporting expression of the cytokine payload. In the reported experiments, treatment promoted dendritic-cell maturation and increased CD8+ T-cell responses. Antitumor effects were also observed beyond the directly treated tumor, suggesting that the immune response was not confined to the local site.

Cytokine delivery has also been explored using mRNA-based systems. Sun et al. (71) designed ALEvs@LNPs for IL-2 expression in triple-negative breast cancer. The platform used an LNP core to carry IL-2 mRNA, while an engineered extracellular-vesicle coating was introduced to improve tumor localization. The system was combined with mild PTT and LAG3 blockade. In the reported model, photothermal treatment altered the local tumor environment, IL-2 expression supported T-cell activation, and LAG3 inhibition reduced T-cell exhaustion. Together, these components improved the response to checkpoint blockade while keeping IL-2 production concentrated at the tumor site. This local expression strategy is important because it may increase cytokine activity in the tumor without exposing the whole body to the same cytokine dose.

Light itself can also be used to control cytokine expression. Lin et al. (90) developed a NIR-II-responsive system based on photothermal conjugated nanoparticles. Heating activated an HSP70 promoter and triggered IL-2 production. In 3D tumor spheroids, local IL-2 levels increased by about 4.3-fold within 30 min after irradiation. The released cytokine was associated with increased IL-2RA expression and activation of NK-92 cells, supporting local tumor cell killing. Other light-responsive strategies work through different mechanisms. Changes in molecular conformation or polarity after light exposure can alter cytokine -receptor interactions or influence the production of immunomodulatory factors. Photoswitchable galactosyl ceramides, for example, have been used under 370 nm light to activate NKT cells and induce cytokines such as IFN-γ (91).

These studies show that PTT can be used not only to generate local heat, but also to influence where and when cytokine activity occurs. This may be useful for cytokines with short half-lives or dose-related systemic toxicity. Local production or light-triggered release can increase cytokine exposure within the tumor while reducing exposure in other tissues. Even so, delivery remains difficult to control. Cytokine payloads may still be released before reaching the tumor, and complex carrier systems can suffer from low loading efficiency or unstable behavior *in vivo*. Most of the current evidence still comes from preclinical models. Before these approaches can move closer to clinical use, their pharmacokinetics, safety, stability, and consistency of cytokine release will need to be examined more carefully.

#### 3.2.5 Photothermal therapy combined with oncolytic virus therapy

Oncolytic viruses (OVs) are natural or engineered viruses that can selectively infect and kill tumor cells. Their antitumor effects come from direct tumor cell lysis and from the release of tumor-associated antigens and inflammatory signals, which can stimulate antitumor immunity (114). However, OV therapy alone often has limited efficacy. Physical barriers in the TME (115), immunosuppression, hypoxia, and poor viral spread within tumors can reduce viral replication and weaken treatment outcomes (116). To address these problems, Huang et al. (92) developed a platelet membrane-coated ICg-OVV composite nanosystem, named PIOVV. This platform combined OV therapy, photodynamic therapy, and PTT. Because oncolytic vaccinia virus (OVV) has relatively high heat tolerance, its viral activity could be maintained under the photothermal conditions used in this system. PTT directly damaged tumor cells and disrupted tumor blood vessels and stromal barriers. This made the tumor tissue more permeable and helped OVV enter deeper tumor regions. After that, OVV-mediated oncolysis further increased tumor cell killing and supported antitumor immune activation. Sakuda et al. (93) combined vesicular stomatitis virus (VSV)-based oncolytic virotherapy with carbon nanotube-mediated photothermal treatment in an osteosarcoma mouse model. Under NIR irradiation, the carbon nanotubes generated local heating at the tumor site. This not only damaged tumor tissue directly, but also appeared to improve intratumoral penetration of VSV. The virus then infected and lysed tumor cells, adding a second mode of tumor killing. In the reported animal experiments, the combined treatment suppressed tumor growth more effectively than either VSV therapy or PTT alone, without an obvious increase in systemic toxicity. Similar combinations have also been tested with other oncolytic viruses. Zhao et al. (94) developed oAd-PPA-IR780, an intranasally administered adenoviral system for glioblastoma. The carrier was designed to respond to the acidic tumor environment, which improved viral transduction, while IR780 provided the photothermal component under NIR irradiation. In an orthotopic brain tumor model, the treatment combined viral infection with local heating and produced a stronger antitumor response than either component used alone. This study illustrates how tumor-responsive polymer engineering can be used to simultaneously improve OV delivery and integrate photothermal treatment. Similarly, Ji et al (95) developed a multifunctional biomimetic platform by co-loading OVV and the NIR-II photothermal/photoacoustic agent IR1061 into *Sophora flavescens*-derived extracellular vesicles (SFOVV@IR1061) for the treatment of diffuse large B-cell lymphoma. The extracellular vesicle-based carrier enhanced tumor-targeted delivery, while IR1061 enabled NIR-II photoacoustic imaging and photothermal therapy. In parallel, OVV-mediated oncolysis promoted further tumor destruction. Importantly, the combined treatment induced immunogenic cell death and enhanced antitumor immune activation, demonstrating that PTT–OV combinations can be integrated with biomimetic delivery and imaging-guided therapy. Together with the OVV- and VSV-based systems described above, these studies highlight the increasing diversity of viral backbones, photothermal agents, and delivery strategies being explored for PTT–OV combination therapy.

An important issue in combining PTT with oncolytic viruses is whether the virus can tolerate the temperature reached during treatment. Heat sensitivity differs across viral species and can also vary with viral structure (117). Vaccinia virus, for example, can retain activity under relatively mild heating conditions (118), whereas enveloped RNA viruses such as VSV may be more sensitive because their surface proteins are vulnerable to thermal stress (119). For this reason, viral infectivity and replication should be examined at the temperatures actually used for PTT rather than assumed to remain unchanged. Thermal compatibility is a key point when PTT is combined with OVs. Different viruses do not respond to heat in the same way. This depends on the viral strain and also on viral structur. The interaction between the two treatments also depends on how heating is applied. Local photothermal damage can loosen tumor tissue and alter stromal barriers, which may improve viral access to regions that are otherwise difficult to reach. Once inside the tumor, the virus can infect and lyse cancer cells and release tumor-associated antigens and damage-related signals. The outcome, however, is likely to depend on several treatment variables, including temperature, heating duration, viral dose, and the interval between virus administration and irradiation. Cooling after treatment may also matter if viral activity is temperature sensitive. In addition, heterogeneous tumor structure and local immunosuppression can restrict viral spread even when the virus performs well under controlled experimental conditions. Direct comparisons across different OV strains and heating regimens would therefore be useful for defining combinations that preserve viral activity while still providing sufficient photothermal damage.

#### 3.2.6 Photothermal therapy combined with bispecific antibodies

Bispecific antibodies (BsAbs) can bind two different epitopes or antigens at the same time. This gives them a different function from conventional monoclonal antibodies. Several BsAbs have already been used in hematological cancers, and some are being tested in solid tumors (120). The reason for combining PTT with BsAbs is relatively straightforward. PTT can heat and damage tumor cells and release TAAs. These antigens may then be recognized by immune cells recruited or activated through BsAb-mediated targeting. In this setting, BsAbs may help redirect immune cells toward tumor cells, while PTT changes the local tumor environment and increases antigen release. Xie et al. (96) developed a nanobody-guided T-cell engager, CD105/CD3 Nb-LipoICG, using PEGylated liposomes as carriers. The liposome surface was modified with CD105/CD3 bispecific nanobodies, and ICG was loaded inside the carrier for photothermal treatment and fluorescence imaging. After NIR irradiation, ICG generated local heat, directly damaged tumor cells, and promoted antigen release. At the same time, the CD105/CD3 bispecific nanobodies activated T cells. Local heating also improved the TME and increased T-cell infiltration. This strategy addressed one common limitation in solid tumor immunotherapy: insufficient effector T-cell entry into tumor tissues. Guo et al. (97) developed a hollow polydopamine nanosphere system, HPDA@MK. The particles were loaded with MK-8628, a bromodomain inhibitor. MK-8628 can suppress c-MYC transcriptional activity and, in this study, was used to reduce PD-L1 and CD47 expression indirectly. After NIR irradiation, HPDA@MK produced a photothermal effect, damaged tumor cells, and promoted TAA release. MK-8628 also increased tumor cell apoptosis and lowered PD-L1 and CD47 levels. In the treated tumors, the authors observed more cytotoxic T-cell proliferation, less M2 macrophage polarization, weaker immunosuppression in the TME, and reduced tumor growth. PTT may also make tumors more responsive to BsAb-based immunotherapy. By inducing ICD and releasing DAMPs, PTT can create a more inflammatory local environment. BsAbs may then help bring immune effector cells closer to tumor cells. Xu et al. (98) used cycloacetylated hollow ruthenium nanoparticles to co-deliver RBT, a fluorescent antitumor ruthenium complex, together with a bispecific antibody, SS-Fc. In this system, SS-Fc served as a bridge between NK cells and tumor cells and improved NK cell-mediated tumor killing.

Several issues still need to be worked out for PTT-BsAb combinations. Treatment order is one of them. In studies (97, 98), PTT was given before BsAb administration, but whether this sequence is optimal for immune-cell recruitment or nanoparticle delivery has not been examined in detail. Mild heating may also produce uneven immunogenic cell death across the tumor. Tumor cells that survive PTT and lack the antigen recognized by the BsAb could remain untreated and later contribute to regrowth. Co-delivery creates a different problem. PTAs and BsAbs have very different physicochemical properties, so it is not easy to make both components reach the tumor together, remain stable, and retain their activity after delivery. A useful carrier would need to preserve the photothermal function of the PTA without compromising antibody binding or bioactivity. Tumor heterogeneity makes this even more difficult, because BsAb activity depends on the presence of the target antigen, and that expression may vary between patients or even between different regions of the same tumor. The therapeutic efficacy of BsAbs depends on the sufficient and relatively uniform expression of target antigens on tumor cells. However, target expression can vary considerably among patients and even within different regions of the same tumor, potentially reducing the effectiveness of combination therapy. A key safety concern in combining PTT with T-cell-engaging BsAbs is cytokine release syndrome (CRS). BsAbs trigger systemic cytokine release via T-cell activation, while PTT adds to local inflammatory through DAMPs and pro-inflammatory mediators. This local thermal response could potentially worsen BsAb-related CRS, a factor that needs dedicated systematic evaluation. Moving PTT -BsAb approaches toward translation will require testing different dosing schemes, sequence protocols, and inflammatory profiles (121). Platform designs might also benefit from altered carrier surfaces or responsive release features. Additionally, clearing up how photothermal heating alters BsAbs distribution, immune recognition, and target binding will be useful for working out the real value of this combination.

## 4 Conclusions and outlook

Although PTT combined with immunotherapy has shown activity against primary tumors and metastases in preclinical studies, moving this strategy into the clinic is still difficult (122). One practical problem is the frequent use of intratumoral injection. This route is hard to apply to deep tumors, multiple lesions, or metastatic disease. For broader use, intravenous delivery will be needed, but this also raises a new problem: the carrier must reach the tumor while avoiding unnecessary immune activation in normal tissues. It should not be assumed that nanocarriers fully protect off-target tissues. If immune regulators such as anti-PD-L1 or anti-LAG-3 are exposed on the particle surface, they may still act in the circulation. Delivery systems that remain relatively inactive in blood and become active mainly in the TME may therefore be more useful.The immune effect of PTT also needs to be defined more carefully. PTT-induced ICD is not equally strong in all tumor models. Mild heating can also increase PD-L1 expression, which partly explains why ICIs are often needed. Still, the best treatment order is not clear. At present, timing is often chosen empirically. Early markers, such as nanoparticle distribution inside tumors or short-term changes in T-cell infiltration, may help decide when to add ICIs and which patients are more likely to respond. For combinations involving OVs, ACT, or cytokines, it will also be important to understand the short period after PTT when tumor vessels become more permeable and immune cells are recruited. This window may decide whether the therapeutic payload enters the tumor or is cleared too quickly.

Future material design should be guided more by biological compatibility than by structural complexity. Many current platforms are elegant, but their long-term safety, degradation, and metabolism are still unclear. Instead of continuously building more complicated multifunctional systems, the field may benefit from simpler and scalable carriers, such as liposomes or endogenous membrane-based platforms, especially when their metabolic fate can be predicted.

To close the gap between preclinical results and clinical use, better models are also needed. Humanized models and patient-derived organoids may provide more realistic information than conventional mouse tumor models. Direct comparison of treatment sequence, dose range, heating conditions, and systemic toxicity across different tumor types will be needed before PTT-based immunotherapy can become a reliable clinical strategy.
